# Phenotypic and Functional Characterization of Double Negative B Cells in the Blood of Individuals With Obesity

**DOI:** 10.3389/fimmu.2021.616650

**Published:** 2021-02-23

**Authors:** Daniela Frasca, Alain Diaz, Maria Romero, Bonnie B. Blomberg

**Affiliations:** ^1^ Department of Microbiology and Immunology, University of Miami Miller School of Medicine, Miami, FL, United States; ^2^ Sylvester Comprehensive Cancer Center, University of Miami Miller School of Medicine, Miami, FL, United States

**Keywords:** aging, B cells, obesity, inflammation, antibody responses

## Abstract

We have previously shown that obesity is associated with increased secretion of IgG antibodies with anti-self-reactivity. In this paper, we confirm and extend our previous findings. We show that the plasma of individuals with obesity is enriched in autoimmune antibodies whose levels are positively associated with blood frequencies of the subset of Double Negative (DN) B cells, which is the most pro-inflammatory B cell subset. We also show that DN B cells, significantly increased in the blood of obese versus lean individuals, are characterized by higher expression of immune activation markers and of the transcription factor T-bet, both associated with autoimmunity. The removal of DN B cells from the peripheral B cell pool significantly decreases *in vitro* secretion of anti-self IgG antibodies. These results altogether confirm the crucial role of DN B cells in the secretion of anti-self IgG antibodies in individuals with obesity.

## Introduction

Obesity, defined as body-mass index (BMI) ≥ 30 kg/m^2^ by the Centers for Disease Control and Prevention and the World Health Organization, is a condition associated with chronic low-grade systemic inflammation, known as inflammaging (1). Inflammaging has been shown to induce chronic immune activation (IA), which contributes to functional impairment of immune cells and decreased immunity. Obesity and associated inflammation lead to several debilitating chronic diseases such as type-2 diabetes, cancer, atherosclerosis, and inflammatory bowel disease (2–9).

We have previously shown that obesity is associated with decreased antibody responses to the influenza vaccine and decreased B cell function (10), measured by activation-induced cytidine deaminase (AID) after *in vivo* or *in vitro* stimulation with mitogens, antigens and vaccines. AID is the enzyme that regulates Ig class switch recombination (CSR) and somatic hypermutation (SHM) (11), two processes leading to the generation of high affinity protective antibodies (12–14). The reduced B cell resposes in individuals with obesity are likely due to the fact that B cells from obese individuals, as compared to those from lean individuals, are enriched in memory B cells, and in particular in the subset of Double Negative (DN) B cells, which is the most pro-inflammatory B cell subset (10, 15), reported to be increased in the blood of individuals with inflammatory conditions and diseases. These include aging (16–18), autoimmune diseases such as Rheumatoid Arthritis (19), Systemic Lupus Erythematosus (SLE) (20, 21), Multiple Sclerosis (22), Alzheimer’s disease (23), Sjogren’s disease (24) and pemphigus (25). DN B cells have also been reported to be increased in the blood of patients affected by chronic infectious diseases such as HIV (26), Hepatitis C (27) and Malaria (28). These results have suggested that these cells likely expand *in vivo* after chronic exposure to autoantigens or pathogen-derived antigens, leading to the production of autoimmune or protective antibodies, respectively. DN B cells are also expanded in the blood of COVID-19 patients and associated with anti-viral antibody responses and poor clinical outcomes, as recently shown (29).

In this paper, we show that the plasma of individuals with obesity is enriched in anti-self IgG antibodies and we tested three different antigenic specificities: double strand (ds)DNA, malondihyldehyde (MDA) and adipocyte-derived antigens. We chose these antigenic specificities because obesity is associated with increased DNA damage (measured by dsDNA) (30), increased oxidative stress and lipid peroxidation (measured by MDA) (31, 32), and increased fat mass (measured by adipocyte-associated antigens released by the adipose tissue) (33). Plasma levels of these anti-self IgG antibodies are positively associated with blood frequencies of DN B cells. We confirmed our previous findings that the frequencies of DN B cells are increased in the blood of obese versus lean individuals. Moreover, we found that DN B cells show higher expression of IA markers and of the transcription factor T-bet associated with autoimmunity. The removal of DN B cells from the total B cell pool significantly reduced *in vitro* secretion of anti-self IgG antibodies. These results reveal a critical role for DN B cells in the secretion of anti-self IgG antibodies in individuals with obesity.

## Materials and Methods

### Subjects

Experiments were performed using blood isolated from lean (n=20, 30–54 years) and obese (n=20, 27–55 years) adult female individuals, with average body Mass Index (BMI, kg/m^2^) 21 ± 1 and 42 ± 3, respectively. The individuals participating in the study were screened for diseases known to alter the immune response or for consumption of medications that could alter the immune response. We excluded subjects with autoimmune diseases, congestive heart failure, cardiovascular disease, chronic renal failure, malignancies, renal or hepatic diseases, infectious disease, trauma or surgery, pregnancy, or documented current substance and/or alcohol abuse.

Study participants provided written informed consent. The study was reviewed and approved by our Institutional Review Board (IRB, protocols 20070481 and 20160542), which reviews all human research conducted under the auspices of the University of Miami.

### PBMC Collection

PBMC were collected using Vacutainer CPT tubes (BD 362761) and cryopreserved. PBMC (1x10^6^/ml) were thawed and cultured in complete medium (c-RPMI, RPMI 1640, supplemented with 10% FCS, 10 µg/ml Pen-Strep, 1 mM Sodium Pyruvate, and 2 x 10^–5^ M 2-ME and 2 mM L-glutamine).

### Flow Cytometry

After thawing, PBMC (2 x 10^6^/ml) were stained for 20 min at room temperature with the following antibodies: anti-CD45 (BioLegend 368540), anti-CD19 (BD 555415), anti-CD27 (BD 555441), and anti-IgD (BD 555778) to measure naive (IgD+CD27-), IgM memory (IgD+CD27+), switched memory (IgD-CD27+), and DN (IgD-CD27-) B cells. To measure membrane expression of markers associated with IA, B cells were also stained with anti-CD95 (BioLegend 305635), anti-CD21 (BioLegend 354911), anti-CD11c (BioLegend 301625), anti-CD86 (BioLegend 374215), anti-HLADR (BioLegend 307617), anti-PD1 (BioLegend 329907) antibodies. Up to 10^4^ events in the B cell gate were acquired on an LSR-Fortessa (BD) and analyzed using FlowJo 10.0.6 software. Single color controls were included in every experiment for compensation. Isotype controls were also used in every experiment to set up the gates.

### B Cell Isolation and Stimulation

After thawing, B cells were isolated from PBMC using magnetic CD19 Microbeads (Miltenyi), following manufacturer’s instructions. Cell preparations were typically >98% pure. B cells were stimulated in c-RPMI with CpG (InvivoGen ODN2006, 10 μg/ml) for 10 days. Supernatants were collected and IgG specificity was measured by ELISA.

To evaluate the effects of DN B cells on IgG autoantibody secretion, CD19+ B cells isolated with magnetic beads were stained with anti-CD27 and anti-IgD antibodies. DN B cells were sorted out in a Sony SH800 cell sorter. Total B cells and total B cells without DN B cells were stimulated for 10 days with CpG, and supernatants analyzed for IgG autoantibody specificity by ELISA.

### RNA Extraction and Quantitative PCR

Total RNA was extracted from unstimulated DN B cells, resuspended in TRIzol, according to the manufacturer’s protocol, then resuspended into 10 µl of preheated H_2_O, and stored at -80°C until use. Reverse Transcriptase (RT) reactions were performed in a Mastercycler Eppendorf Thermocycler to obtain cDNA. Briefly, 2 µl of RNA at the concentration of 0.5 µg/µl were used as template for cDNA synthesis in the RT reaction. Conditions were: 40 min at 42°C and 5 min at 65°C. Five µl of cDNA were used for qPCR. Reactions were conducted in MicroAmp 96-well plates and run in the ABI 7300 machine. Calculations were made with ABI software. Briefly, we determined the cycle number at which transcripts reached a significant threshold (Ct) for each target gene and for GAPDH as control. A value of the target gene, relative to GAPDH, was calculated and expressed as ΔCt. Reagents and primers (Taqman) were from ThermoFisher.

### ELISA to Measure Antibodies in Plasma and Culture Supernatants

For dsDNA-specific and Malondihyldehyde (MDA)-specific IgG antibodies we used the Signosis EA-5002 and MyBioSource MBS390120 kits, respectively.

For adipocyte-specific IgG antibodies, we isolated the adipocytes from the subcutaneous adipose tissue of patients undergoing weight reduction surgeries (bilateral breast reduction), as previously described (33). After isolation, the adipocytes were centrifuged in a 5415C Eppendorf microfuge (2,000 rpm, 5 min). Total cell lysates were obtained using the M-PER (Mammalian Protein Extraction Reagent, ThermoFisher), according to the manufacturer’s instructions. Aliquots of the protein extracts were stored at -80°C. Protein content was determined by Bradford (34).

### Statistical Analyses

To examine differences between groups, unpaired Student’s t tests (two-tailed) were used. To examine relationships between variables, bivariate Pearson’s correlation analyses were performed, using GraphPad Prism version 8 software, which was used to construct all graphs. Principal Component Analyses (PCA) were generated using RStudio Version 1.1.463.

## Results

### The Plasma of Individuals With Obesity Is Enriched in IgG Antibodies Specific for dsDNA, MDA, and Adipocyte-Derived Antigens

**Figure 1:**
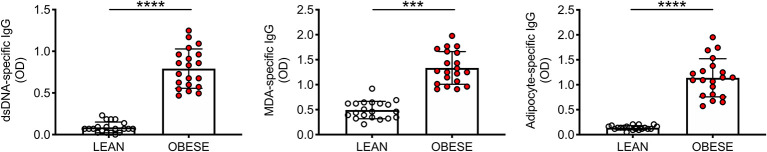
The plasma of individuals with obesity is enriched in IgG antibodies specific for dsDNA, MDA and adipocyte-derived antigens. Plasma samples were isolated from individuals with obesity and from lean controls. Mean comparisons between groups were performed by Student’s t test (two-tailed). ***p < 0.001, ****p < 0.0001.

Plasma samples were isolated from individuals with obesity and from lean controls. Samples were tested for the presence of IgG antibodies specific for ds-DNA, MDA and adipocyte-derived antigens. **Figure 1** shows significantly higher amounts of IgG for the 3 different antigenic specificities in obese versus lean individuals.

We also measured IgM antibodies specific for the above autoantigens. Results show no significant differences in lean versus obese individuals for anti-ds-DNA IgM antibodies (0.84 ± 0.11 *vs.* 0.93 ± 0.13, p=0.60, n=6), for MDA IgM antibodies (1.39 ± 0.09 *vs.* 1.58 ± 0.08, p=0.15, n=6), and for IgM specific for adipocyte-derived antigens (1.26 ± 0.13 *vs.* 1.33 ± 0.09, p=0.06, n=18).

### The Frequencies of DN B Cells Significantly Increase in the Blood of Obese Versus Lean Individuals

We have previously shown that DN B cells present in the blood and in the adipose tissue of individuals with obesity are responsible for the secretion of anti-adipocyte-specific IgG antibodies (15). Here, we tested the hypothesis that DN B cells were also associated with/responsible for the secretion of anti-dsDNA and anti-MDA IgG antibodies in the blood of obese individuals.

**Figure 2:**
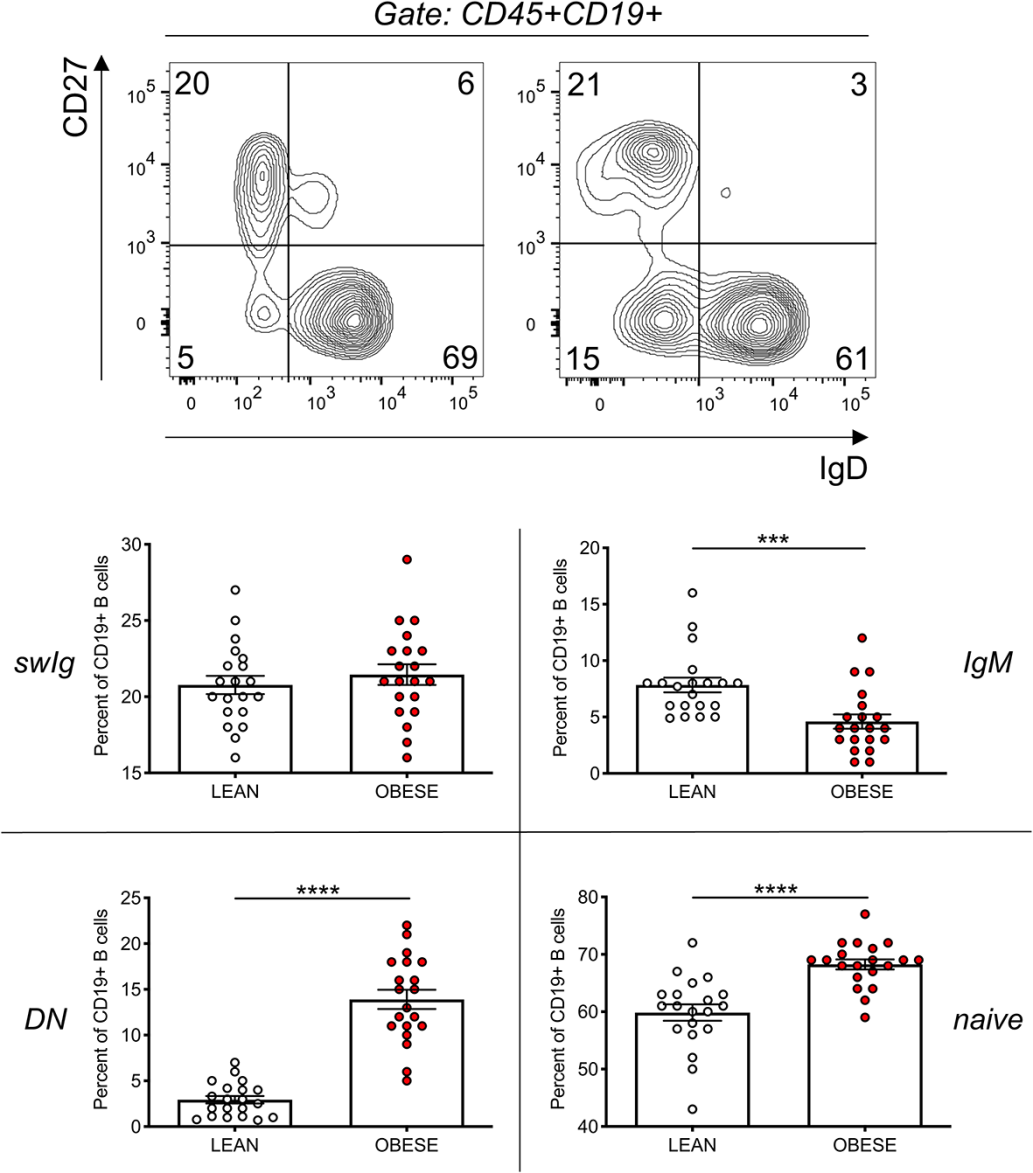
The frequencies of DN B cells significantly increase in the blood of obese versus lean individuals. Top. Gating strategies and a representative dot plot from one lean and one obese individual. Bottom. Results show frequencies of the four B cell subsets. Mean comparisons between groups were performed by Student’s t test (two-tailed). ***p < 0.001, ****p < 0.0001.

We therefore compared the frequencies of DN B cells in this cohort of obese and lean individuals. **Figure 2** (top) shows the major B cell subsets, gated on leukocytes (CD45+): naive (IgD+CD27-), IgM memory (IgD+CD27+), switched memory (swIg, IgD-CD27+) and DN (IgD-CD27-). Results in **Figure 2** (bottom) show the significant increase in the frequencies of DN B cells in obese versus lean individuals, confirming and extending to this cohort our previously published findings (10, 15). Results in **Figure 2** (bottom) also show the frequencies of the other B cell subsets. We observed a significant increase in the frequencies of naïve and a significant decrease in the frequencies of IgM memory B cells in obese versus lean individuals, whereas the frequencies of swIg were found not significantly different between the two groups. These results are slightly different from those we have previously published (10), likely because in this study we have included individuals that are older (27–55 years) than those in our previous study (20–40 years).

### IgG Antibodies Specific for dsDNA, MDA, Adipocyte-Derived Antigens Are Positively Associated With Blood Frequencies of DN B Cells

**Figure 3:**
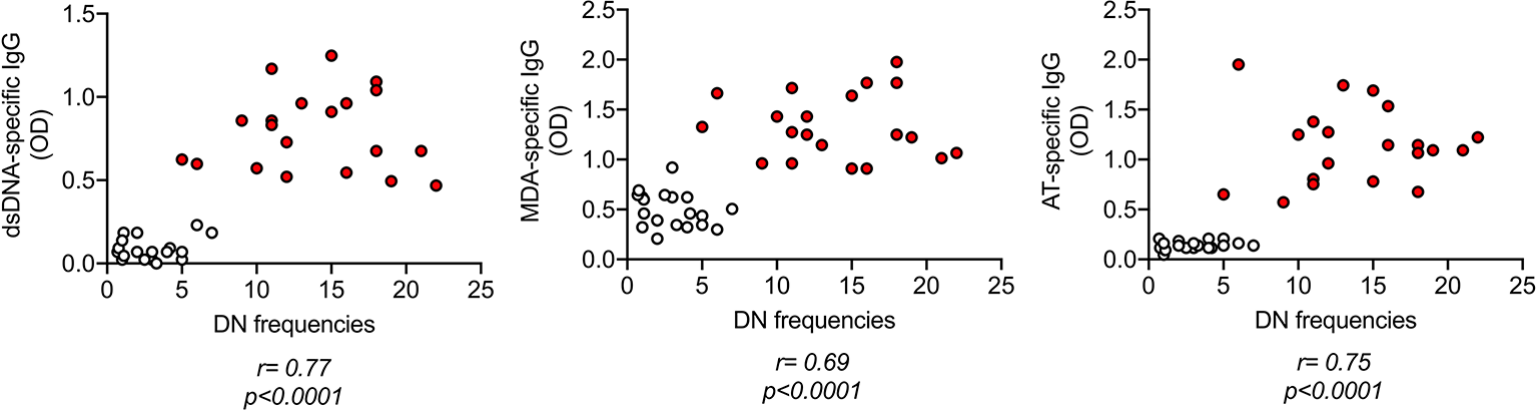
IgG antibodies specific for dsDNA, MDA, adipocyte-derived antigens are positively associated with blood frequencies of DN B cells. IgG antibodies were measured in plasma as indicated in **Figure 1**. DN B cell frequencies were measured by flow cytometry as indicated in **Figure 2**. Correlation coefficients and p values are shown for each antibody specificity.

As expected, IgG antibodies specific for the self-antigens in **Figure 1** were positively associated with blood frequencies of DN B cells in **Figure 2** (**Figure 3**).

#### DN B Cells Are Characterized by Higher Expression of IA Markers Associated With Autoimmunity

**Figure 4:**
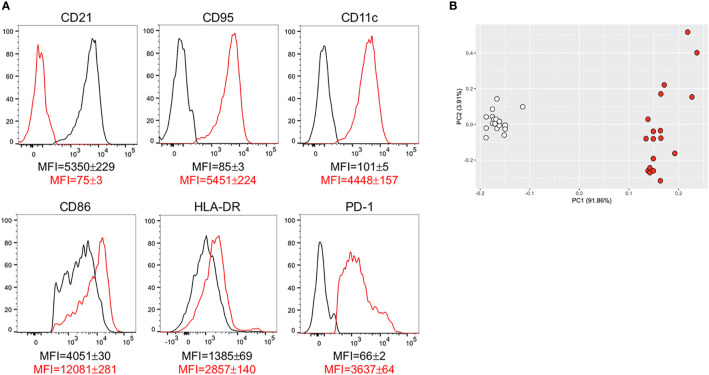
DN B cells are characterized by higher expression of IA markers associated with autoimmunity. **(A)** Cells were stained to evaluate the expression of several markers of IA on DN B cells from individuals with obesity and from lean controls. Results show mean fluorescence intensity (MFI)± SE for each marker in DN B cells from lean (black line) and obese (red line) individuals (18 individuals/group). **(B)** PCA analysis with the axes showing the percentage of variation explained by PC1 and PC2. Each symbol indicates an individual. White symbols: lean individuals. Red symbols: obese individuals.

In order to characterize the phenotype of DN B cells present in the blood of individuals with obesity and of lean controls, we examined membrane expression of markers of IA, previously shown to be present on DN B cells from patients with autoimmunity. Briefly, we measured the following: CD21, the complement receptor for C3d (35); CD95, Fas ligand (36); CD11c, the Itgax integrin involved in antigen presentation to T cells (37); CD86 and HLADR, also involved in antigen presentation to T cells (38, 39); PD1, a marker of IA and of cell exhaustion (40). Results in **Figure 4A** show that DN B cells from individuals with obesity are characterized by lower levels of expression of CD21, and higher levels of expression of CD95, CD11c, CD86, HLADR, PD1, as compared to those from lean controls. These results are in agreement with previously published observations showing the association of the membrane phenotype CD21^low^CD95+CD11c+CD86+HLADR+PD1+ with autoimmune B cell subsets, and clearly demonstrate that obesity induces the expansion of DN B cells characterized by this autoimmune phenotype. In the PCA analysis in **Figure 4B** distinct clustering of DN B cells from the two groups of individuals are shown.

### DN B Cells Are Also Characterized by Higher Expression of the Transcription Factor T-Bet Associated With Autoimmunity

**Figure 5:**
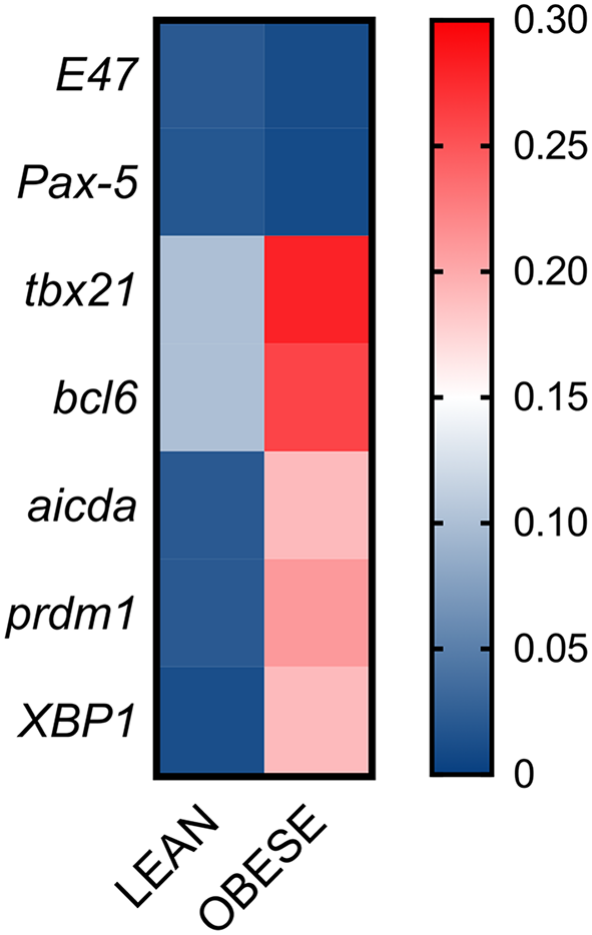
DN B cells are characterized by higher expression of transcription factors associated with autoimmunity. DN B cells were sorted from the peripheral blood of individuals with obesity and of lean controls and left unstimulated. Total RNA was extracted to evaluate by qPCR the expression of transcription factors. Heatmap shows qPCR values (2^-ΔΔCt^) of several transcription factors, normalized to GAPDH. Results show average qPCR values from 18 individuals/group.

Next, we evaluated if DN B cells with the membrane phenotype associated with autoimmunity were expressing not only the transcription factor T-bet, known to be involved in the secretion of anti–self-antibodies, but also the expression of transcription factors and enzymes crucial for CSR. Briefly, we measured RNA expression of T-bet (tbx21) and other transcription factors involved in CSR (E47, Pax-5), in germinal center reactions (bcl6), in plasma cell differentiation (prdm1, XBP1), as well as RNA expression of AID (aicda). Results in **Figure 5** show that tbx21, bcl6, aicda, prdm1 and XBP1 are all significantly up-regulated in unstimulated DN B cells from individuals with obesity as compared to lean controls. No differences were observed for E47 and Pax-5. These results show that DN B cells isolated from the blood of individuals with obesity, as compared to those isolated from lean controls, are not only already pre-activated, as indicated by their higher expression of IA markers, but also show spontaneous expression of the transcription factors associated with antibody secretion, including T-bet, associated with the secretion of IgG antibodies with anti–self–specificity.

### The Removal of DN B Cells Significantly Reduces the Secretion of IgG Autoimmune Antibodies

**Figure 6:**
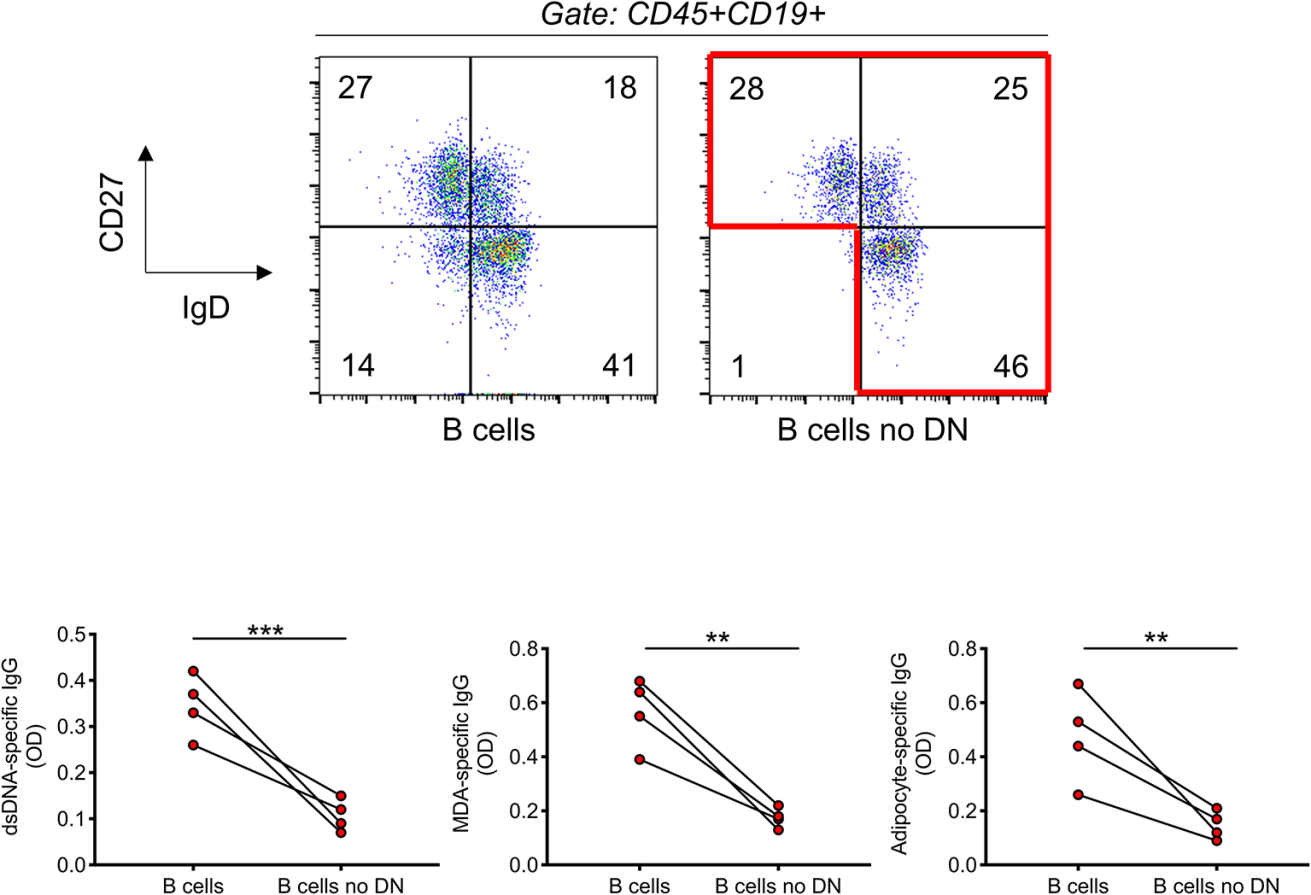
The removal of DN B cells significantly reduces the secretion of IgG autoimmune antibodies. B cells were isolated with magnetic beads from the blood of four individuals with obesity. Top. Gating strategies to remove DN B cells. B cells were stained with anti-CD27 and anti-IgD antibodies. DN B cells were sorted out using a Sony SH800 cell sorter. Bottom. After stimulation of total B cells and total B cells without DN B cells for 10 days with CpG, supernatants were collected and analyzed for the presence of anti-dsDNA, anti-MDA, and anti-adipocyte IgG by ELISA. **p < 0.01, ***p < 0.001.

We have previously shown that DN B cells sorted from the breast adipose tissue of obese female patients undergoing weight reduction surgeries secrete autoimmune IgG antibodies that are specific for adipocyte-derived antigens (15). These experiments have been possible because from surgery patients we get large pieces of discarded tissue and, also, because DN B cell frequencies in the adipose tissue reach up to 80% of the total B cell pool, a frequency never observed in the peripheral blood. To further confirm that DN B cells are responsible for the secretion of autoimmune IgG antibodies in the blood of individuals with obesity, we performed the following experiment. B cells, as well as B cells without DN B cells, isolated from the blood of individuals with obesity, were stimulated for 10 days with the B cell mitogen CpG. Stimulation is necessary to allow the stimulation/expansion of IgG secreting B cells. After stimulation, supernatants were collected and IgG autoimmune antibodies measured by ELISA. Results in **Figure 6** show that the removal of DN B cells from the pool of total B cells of obese individuals significantly decreased *in vitro* secretion of anti-dsDNA, anti-MDA and anti-adipocyte IgG specific antibodies.

## Discussion

The subset of DN B cells has been the focus of increasing interest in the last few years, as shown by a large number of dedicated publications. DN B cells expand in healthy aging, in autoimmune diseases, in chronic and acute infections. DN B cells also increase in the blood of individuals with obesity and reach significantly high frequencies in the obese subcutaneous adipose tissue, where they secrete large amounts of autoimmune antibodies with different specificities. As we have recently demonstrated, these specificities include adipocyte-derived products, mainly cell-associated proteins and nucleic acids, not known as autoantigens but released in large amounts in the obese adipose tissue under conditions associated with hypoxia and cell death (41). The finding that anti-dsDNA, anti-MDA and anti-adipocyte specific antibodies are increased in the plasma of healthy elderly individuals (15, 42) and obese individuals has suggested that obesity may accelerate age-associated B cell defects. Fat mass indeed increases with age in humans (43, 44) and this is associated with increased inflammaging (1), metabolic dysfunction (5, 45) and development of insulin resistance which also increases with age (46). Moreover, an age-associated increase in the ectopic deposit of triglycerides in several tissues (liver, muscle, heart, pancreas, kidney) (47–51) and in blood vessels (52) occurs, and this is associated with the development and/or progression of age-associated diseases.

Data herein clearly show that DN B cells from individuals with obesity express higher levels of membrane markers of IA associated with autoimmunity as compared to lean controls and are characterized by the phenotype CD21^low^CD95+CD11c+CD86+HLADR+PD1+. They also spontaneously express higher RNA levels for transcription factors involved in the secretion of autoimmune antibodies (tbx21, prdm1, XBP1), suggesting that DN B cells from obese individuals are already pre-activated, a status leading to spontaneous secretion of autoimmune antibodies, as shown in autoimmune diseases (53), and in the obese adipose tissue at least for some specificities (33, 41). Because the IA phenotype of DN B cells from obese individuals is associated with increased energy demands, DN B cells engage in robust metabolic reprogramming to generate sufficient energy to fuel these demands and support autoantibody secretion (15).

Human DN B cells have many similarities with mouse splenic Age-associated B Cells (ABCs) (54, 55), identified as CD19+AA4.1-CD43-CD21-CD23- cells (54–56). DN B cells and ABCs originate from mature B cell subsets (naïve in humans, follicular B cells in mice) after *in vivo* or *in vitro* stimulation with the Toll-like receptors TLR7 or TLR9, alone or together with BCR cross-linking, demonstrating that BCR is also an active signaling system in these subsets. It has been shown that TLR agonists plus IL-21 and IFN-γ regulate T-bet expression, the transcription factor for the secretion of autoimmune antibodies (57), whereas TLR agonists plus IL-21 alone promote CD11c expression independently of T-bet (58). In agreement with the expression of T-bet, both human DN B cells and mouse ABCs secrete anti-ds-DNA (our results herein) or anti-chromatin (55) autoimmune IgG antibodies. Moreover, T-bet+ ABCs carry somatically mutated Ig, suggesting that they originate during T-dependent B cell responses (59). T-bet+ ABCs appear and persist indefinitely after influenza infection in mice (58, 59). These cells represent the spleen-resident population of memory B cells responsible for the secretion of HA stalk-specific IgG2c antibodies and of durable neutralizing antibodies (60). Previous results from Swain’s group have also demonstrated that mouse ABCs are specific for a live influenza virus (A/PR8/34) and these influenza-specific ABCs differentiate into antibody-secreting cells, some of which home to the bone marrow and to the lungs where they persist for months, suggesting their role in providing significant protection (61). Human T-bet+ B cells also have also recently been shown to mediate influenza-specific humoral memory (60). Similar to mouse T-bet+ ABCs, they have an activated phenotype, they are spleen-resident and secrete HA-specific IgG1 antibodies recognizing H1 or H3 viral strains. IgG1 antibodies represent the equivalent of mouse IgG2c.

DN B cells are heterogeneous with two major subsets, DN1 and DN2. DN1 B cells are exclusively involved in follicular T-dependent antibody responses. DN2 B cells, conversely, represent the DN B cell subset that participates in extra-follicular B cell responses. DN1 B cells represent the major DN B cell subset in healthy individuals, whereas DN2 B cells increase in the blood of SARS-CoV-2-infected patients as compared to uninfected controls, suggesting a pathogenic role of DN2 B cells in COVID-19 patients (29). DN2 B cells also increase in the blood of SLE patients, as shown by the same group (62). In both cases, DN2 B cells are characterized by decreased expression of the chemokine receptor CXCR5, associated with follicular homing predisposition, and by a concomitant increased expression of CXCR3, a marker of homing to inflamed tissues. We haven’t been able to identify DN1 and DN2 B cells in the blood and in the adipose tissue of individuals with obesity, likely because the individuals recruited in our studies are healthy, and either acute infection (COVID-19) or active disease (SLE) may be needed to allow the expansion of these extra-follicular B cells. We believe that the DN B cells in obese individuals are predominantly DN2, as they secrete autoimmune antibodies as observed in SLE patients.

In conclusion, our results confirm and extend our previous findings showing that frequencies of DN B cells increase in the blood of obese as compared to lean individuals and are positively correlated with the amounts of plasma autoimmune IgG antibodies. DN B cells are characterized by higher expression of IA markers and of the transcription factor T-bet, both associated with autoimmunity. When we removed DN B cells from the B cell pool we saw a significant decrease in the *in vitro* secretion of anti-self IgG antibodies. We believe that the results herein strongly support the role of DN B cells in the secretion of anti-self IgG antibodies in individuals with obesity.

## Data Availability Statement

The raw data supporting the conclusions of this article will be made available by the authors, without undue reservation.

## Ethics Statement

The studies involving human participants were reviewed and approved by institutional review board (IRB) protocol 20070481 and 20160542. The patients/participants provided their written informed consent to participate in this study.

## Author Contributions

DF wrote the paper. AD, MR, and DF performed the experiments and acquired and analyzed data. DF and BB were involved in funding acquisition. All authors contributed to the article and approved the submitted version.

## Funding

This study was supported by NIH awards AG32576, AG059719, and AG023717.

## Conflict of Interest

The authors declare that the research was conducted in the absence of any commercial or financial relationships that could be construed as a potential conflict of interest.
